# Convergent Evolution by Cancer and Viruses in Evading the NKG2D Immune Response

**DOI:** 10.3390/cancers12123827

**Published:** 2020-12-18

**Authors:** Richard Baugh, Hena Khalique, Leonard W. Seymour

**Affiliations:** Anticancer Viruses and Cancer Vaccines Research Group, Department of Oncology, University of Oxford, Oxford OX3 7DQ, UK; richard.baugh@oncology.ox.ac.uk (R.B.); hena.khalique@oncology.ox.ac.uk (H.K.)

**Keywords:** NKG2D ligands, NKG2D receptor, NK cells, immune evasion, convergent evolution, cancer, viruses, immunotherapy

## Abstract

**Simple Summary:**

Cells undergoing stress, viral infection, and malignant transformation express natural killer group 2 member D (NKG2D) ligands on their surface, rendering them susceptible to immunosurveillance. Given this selective pressure exerted on viruses and cancer cells, many viruses and several cancers have evolved means of evading NKG2D recognition. This review highlights the various ways in which stresses, viruses and cancers induce the expression of NKG2D ligands, before comparing the similarities and differences between viral and cancer mechanisms to subsequently prevent recognition by the NKG2D system.

**Abstract:**

The natural killer group 2 member D (NKG2D) receptor and its family of NKG2D ligands (NKG2DLs) are key components in the innate immune system, triggering NK, γδ and CD8^+^ T cell-mediated immune responses. While surface NKG2DL are rarely found on healthy cells, expression is significantly increased in response to various types of cellular stress, viral infection, and tumour cell transformation. In order to evade immune-mediated cytotoxicity, both pathogenic viruses and cancer cells have evolved various mechanisms of subverting immune defences and preventing NKG2DL expression. Comparisons of the mechanisms employed following virus infection or malignant transformation reveal a pattern of converging evolution at many of the key regulatory steps involved in NKG2DL expression and subsequent immune responses. Exploring ways to target these shared steps in virus- and cancer-mediated immune evasion may provide new mechanistic insights and therapeutic opportunities, for example, using oncolytic virotherapy to re-engage the innate immune system towards cancer cells.

## 1. Introduction

Cancer cells have long been defined with a set of characteristics, known as the ‘hallmarks of cancer’, with immune evasion rapidly emerging as a key player in cancer progression [1]. Viruses share several of these hallmarks with cancer cells to achieve a successful infection, as immune evasion is also critically important for viruses.

### 1.1. NKG2D Receptor and NK Cell Activation

**Figure 1:**
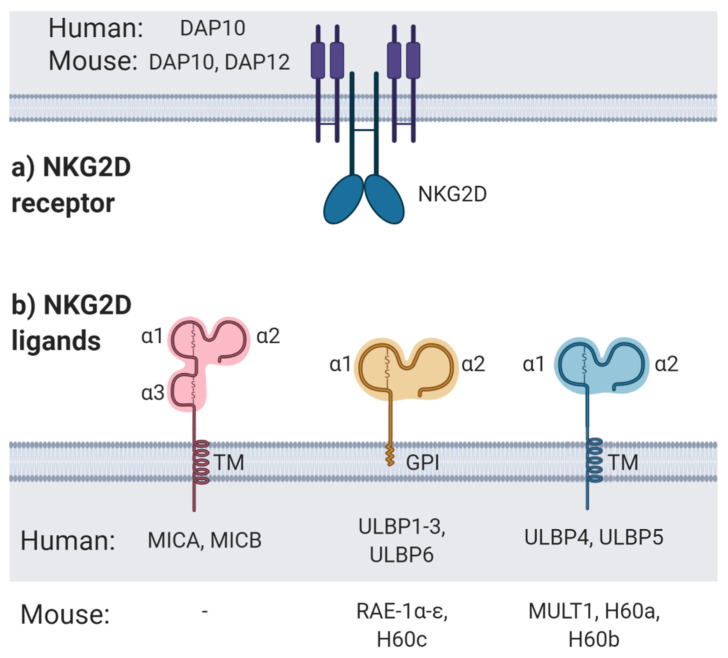
NKG2D receptor and ligands. (**a**) The natural killer group 2 member D (NKG2D) receptor is expressed by natural killer (NK) cells, γδ T cells, CD8^+^ T cells, and some CD4^+^ T cells. The NKG2D receptor is associated with DNAX activating protein (DAP) 10 in humans, but DAP10 and DAP12 in mice. (**b**) NKG2D ligands (NKG2DLs) include MHC-class-I-polypeptide-related sequence (MIC) A and MICB, and UL16 binding protein (ULBP) 1-6 in humans, and retinoic acid early inducible (RAE-1) α-ε, H60a-c, and murine UL16 binding protein-like transcript (MULT) 1 in mice. All NKG2DLs feature MHC-class-I-like α1 and α2 domains, whilst MICA and MICB also have an α3-like domain but do not bind to β2-microglobulin. Some NKG2DLs are transmembrane proteins (TM), while others are linked to the membrane via glycophosphatidylinositol (GPI) anchors. Diagram created with BioRender.com.

Natural killer (NK) cells play a critical role in the innate immunosurveillance of both virally infected cells and tumours. They possess an extensive repertoire of both activatory and inhibitory receptors that recognise a range of ligands on target cells [2]. In contrast to T cell activation by clonotypic T cell receptor (TCR) interacting with specific peptides presented in the context of major histocompatibility complex (MHC), NK cells are inhibited by MHC molecules, and are instead activated by the lack of MHC, referred to as ‘missing-self’ [3]. NK cells also interpret other positive and negative signals from their environment and the target cell. Some of the activatory receptors responsible for processing positive signals include NKp46, NKp44 and NKp30 [2]. One of the most well-documented activatory receptors is the natural killer group 2 member D (NKG2D) receptor. NKG2D receptor and its ligands (NKG2DLs) are part of the early warning signals for the innate immune system; flagging up cells that may represent danger to the host organism. NKG2D receptor is an activatory receptor expressed on the surface of NK cells, CD8^+^ T cells, natural killer T (NKT) cells, γδ T cells and some CD4^+^ T cell subsets [4,5,6]. NKG2D is an invariant homodimeric receptor, with each monomer consisting of a type-II transmembrane domain and a C-type lectin-like extracellular domain, and is associated with DNAX activating protein 10 (DAP10) or DAP12 in mice, and DAP10 only in humans [4] (Figure 1a).

NKG2D binding to NKG2DLs can directly activate NK cells that have already been stimulated with interleukin (IL) -2 or IL-15 [7,8], whereas additional costimulatory signals are required to activate T cells and freshly isolated NK cells [9,10]. Binding of NKG2D to its ligands recruits phosphatidylinositol 3-kinase (PI3K) and growth factor receptor-bound protein 2 (GRB2), triggering a subsequent phosphorylation cascade. If the balance of the overall signalling favours NK cell activation, it can stimulate effector functions including cytokine release and perforin/granzyme-mediated cytotoxicity [11].

### 1.2. NKG2D Ligands

Although the NKG2D receptor is germline-encoded, it can recognise a diverse range of MHC-class-I-related ligands. In humans, these NKG2DLs include MHC-class-I-polypeptide-related sequence A (MIC) A and MICB, and UL16 binding protein (ULBP) 1-6, also known as retinoic acid early inducible transcript 1 (RAET1) proteins [6,12,13,14,15,16,17]. In contrast, murine NKG2DLs include retinoic acid early inducible (RAE-1) α-ε, H60a-c, and murine UL16 binding protein-like transcript (MULT) 1 [18,19]. All NKG2DLs share homology with MHC class I molecules, featuring α1 and α2 domains, while MICA and MICB also have an α3 domain. However, unlike MHC class I molecules, they cannot bind and present antigenic peptides and are not associated with β_2_-microglobulin. Despite their overall similarities, NKG2DLs vary from each other in sequence; binding affinity to NKG2D; and membrane anchorage, either transmembrane proteins or glycophosphatidylinositol (GPI)-linked [20] (Figure 1b). NKG2DLs also display a high degree of polymorphism in humans, with 104 and 37 distinct alleles currently assigned for MICA and MICB genes, respectively [21]. These polymorphisms can vary certain properties of the NKG2DLs, such as binding affinity to NKG2D receptor [22,23] or protein length [24,25].

The expression of NKG2DLs on normal, healthy cells is low or absent. However, expression dramatically increases during events of cellular stress, virus infection or malignant transformation. This expression pattern in stressed, virus-infected or cancer cells is often referred to as ‘induced self’; whereby germline-encoded NKG2DLs are upregulated, enabling NK or T cell recognition, and often occurs in parallel with viral- or cancer-mediated downregulation of MHC class I expression (i.e., ‘missing-self’) [26]. Upregulation of NKG2DLs allows for rapid NK or T cell-mediated immunosurveillance and elimination of cells that may pose as a threat to the organism: cells that have undergone DNA damage after heat or oxidative shock, and may therefore have acquired oncogenic mutations; cells that have been infected by a viral pathogen, and may lead to a larger-scale infection if left unchecked; or rapidly proliferating cells that may progress to become cancer.

### 1.3. Evading Detection by NKG2D

In the constant battle between the host immune system and cancer or viruses, a plethora of mechanisms are employed by both cancer cells and virus-infected cells to counteract the NKG2D-mediated immune response. A detailed insight into regulatory mechanisms for NKG2DL expression is reviewed by Raulet et al. [20]. This review covers in detail the various mechanisms in which stresses, viruses and cancers can induce the expression of NKG2DLs, before discussing the strategies employed by viruses and cancers to subsequently evade the NKG2D response. Finally, this review highlights the converging evolution displayed by both viruses and cancers to target the critical stages in its regulation.

## 2. NKG2DL Expression in Healthy Cells and Autoimmunity

NKG2DL expression can lead to NK and T cell-mediated cytotoxicity and inflammation. Therefore, surface expression of these ligands is minimal in healthy quiescent cells to prevent autoimmunity. For example, although MICA is expressed at a low level by healthy intestinal epithelial cells (IECs), patients with Crohn’s Disease demonstrate increased MICA expression on these IECs compared to healthy controls, leading to activation and autoimmunity from a subset of CD4^+^NKG2D^+^ T cells [5]. Interestingly, Schrambach and colleagues revealed MICA and MICB mRNA transcripts in healthy organ tissue as well as various tumours [27], suggesting regulation of surface NKG2DL expression is not solely controlled at a transcriptional level.

### 2.1. Cell Stress Induces NKG2DL mRNA and Protein Expression

NKG2DL expression in normal cells is increased following a variety of cellular insults. Human IECs exposed to heat shock demonstrated increased MICA and MICB mRNAs and surface protein expression, along with an increase in heat shock protein (hsp) 70 mRNA [28]. The heat shock-induced upregulation of MICA and MICB also sensitised the target cells to lysis by γδ T cells [28]. A study using human colorectal carcinoma cell lines demonstrated that MICA and MICB mRNA and surface protein expression were low when cells were at high confluency and quiescent. However, the expression of both mRNAs and surface protein was greatly increased in response to oxidative or heat stress, or when cells were proliferating [29]. This stress response is thought to be mediated, at least initially, at the level of transcription, since the promoter regions for MICA and MICB contained conserved heat shock elements (HSE), which are capable of binding heat shock factor 1 (HSF1) in response to heat shock and oxidative stress in a manner similar to regulation of hsp70 expression [12,29]. In a similar study performed on the human colorectal carcinoma cell line CaCo-2, oxidative stress induced by H_2_O_2_ also increased MICA and MICB mRNA expression, although surface protein levels were not measured [30].

Other forms of cell stress have also been reported to induce NKG2DL expression. Endoplasmic reticulum (ER) stress, induced with an inhibitor of the ER Ca^2+^ pump, increased ULBP1, 2, 5, and 6 mRNAs and surface protein in a range of human intestinal epithelial cell lines, as well as MULT1 in murine cells in vivo [31].

DNA damage is another form of cellular stress capable of upregulating surface NKG2DL expression. Gasser et al. demonstrated that activation of the DNA damage response (DDR) by chemotherapeutic agents, ionising radiation or stalling of DNA replication cycles increased NKG2DL expression. This increased NKG2DL expression was found to occur in an ataxia telangiectasia, mutated- (ATM), ATM- and Rad3-related- (ATR) and checkpoint kinase (Chk) 1-dependent manner, sensitising cells to lysis by NK cells [32]. Further evidence towards the link between reactive oxygen species, DNA damage and NKG2DL upregulation was demonstrated by using sublethal doses of chemotherapeutic agents which cause oxidative stress, such as doxorubicin and melphalan, which triggered MICA upregulation in multiple myeloma cells. This transcriptional increase in MICA was attributed to DDR-dependent activity of the transcription factor E2F1 [33]. Additionally, the *ULBP1* and *ULBP2* genes have a response element for the tumour suppressor protein p53, which is stabilised during the DDR. Hence p53 stabilisation during the DDR directly causes an increase in ULBP1 and ULBP2 transcription [34,35].

### 2.2. Post-Transcriptional Regulation of NKG2DL Expression During Cell Stress

Protein expression is not only controlled at the level of transcription; mRNA stability, protein stability and intracellular localisation also play a significant role in regulating functional protein expression. As mentioned earlier, Schrambach et al. observed that MICA and MICB mRNA transcripts were expressed in various healthy human tissues [27], which appears in contrast to the concept that NKG2DL proteins are not expressed by healthy cells, indicating that other regulatory mechanisms are involved beyond gene transcription.

Interestingly, Vantourout et al. describe a mechanism in which ultraviolet B (UVB) radiation upregulated MICA, MICB and ULBP2 in human epithelial cells via stress-induced epidermal growth factor receptor (EGFR) signalling, rather than due to the DDR [36]. They found that under normal conditions, AU-rich element/poly(U)-binding/degradation factor 1 (AUF1) protein targets AU-rich elements (AREs) in the 3′ untranslated region (UTR) of human NKG2DL mRNAs. AUF1 binding to NKG2DL transcripts causes mRNA destabilisation and degradation. However, stress-induced EGFR signalling prevents AUF1 binding and NKG2DL mRNA destabilisation, thus, allowing translation and NKG2DL protein expression.

MicroRNAs (miRNAs) have also been implicated in the regulation of many genes, including MICA and MICB. A particular set of miRNAs found to be expressed in normal human cells can bind to the 3′ UTR of MICA and MICB mRNA transcripts, resulting in their destabilisation and degradation, hence preventing protein translation [37]. It has been hypothesised that these miRNAs play a critical part in the regulation of MICA and MICB protein expression and preventing unwanted autoimmunity. During normal conditions, these miRNAs are expressed, establishing a threshold for MICA and MICB mRNA to reach for protein expression and NKG2D recognition and subsequent cell lysis. During transient cell stress, such as heat shock, the MICA and MICB mRNA levels dramatically increase, while the miRNA expression remains relatively unchanged, enabling a saturation of the miRNAs and for some MICA/B mRNA transcripts to escape miRNA-mediated degradation, and thus, allow protein translation. It has been speculated that this system endows several advantages, such as rapid increases in protein expression, while preventing NKG2D recognition of otherwise healthy cells, due to small fluctuations in MICA or MICB expression [37].

In contrast to the findings regarding p53-mediated increase in *ULBP1* and *ULBP2* transcription mentioned earlier [34,35], p53 also induces expression of miR-34a and miR-34c, which target ULBP2 mRNA for destabilisation [38]. These observations suggest two possibly contrasting roles for p53 in NKG2DL expression and requires more investigation into how the regulation is fine-tuned.

Additionally, healthy primary human bronchial epithelial cells constitutively expressed NKG2DL mRNA transcripts but lacked surface protein expression. However, increased surface NKG2DL expression was detected upon exposure to oxidative stress in the form of H_2_O_2_, although the mRNA and total protein levels remained consistent, indicating a stress-mediated activation of protein translocation to the surface [39]. This rapid method of protein translocation and increased surface expression may allow quicker responses and immunological detection of oxidatively-stressed cells within the well-oxygenated pulmonary environment.

Protein stability has also been reported to play a role in regulating murine NKG2DL expression [40]. Nice et al. report that during normal conditions, MULT1 is targeted by membrane-associated RING-CH (MARCH) 4 and MARCH9 E3 ubiquitin ligases and is subsequently degraded. Upon heat shock treatment, however, this degradation was inhibited, and surface MULT1 increased [40].

## 3. NKG2DL Expression Is Induced in Virally Infected Cells

Viral infection of a cell and the subsequent virus-mediated change of the cellular phenotype to enable viral genome replication, immune evasion and proliferation leads to a highly stressful environment for the host cell. Viral genomic replication [41,42,43], production of viral proteins [42,44], altering cellular metabolism [45], and manipulating signalling pathways to support the infection [46,47,48] are all common traits shared by many viruses during infection, all of which can induce some degree of stress to the host cell. Therefore, the process of viral infection is likely to trigger many of the cellular stresses mentioned in the previous section, such as DDR activation [41], oxidative stress [49,50], and heat shock [51], which would induce the expression of NKG2DLs. However, as described below, viruses also employ a variety of mechanisms to subsequently downregulate these ligands.

As mentioned earlier, the DDR is one of the major regulators of NKG2DL expression in stressed cells, but it is also heavily involved in upregulation of NKG2DL expression in virus-infected cells. Many viruses, especially double-stranded DNA (dsDNA) viruses, have been demonstrated to trigger the DDR during early stages of infection. Some examples include herpes simplex virus (HSV) [52], Epstein-Barr virus (EBV) [43], adenovirus [53], and human immunodeficiency virus (HIV) [42]. Given that DDR activation induces NKG2DL expression [32], viruses that do trigger the DDR during early stages of virus infection subsequently induce NKG2DL expression [54]. Furthermore, if left unrestrained, the DDR would likely concatemerise the nascent virus DNA genomes together. Accordingly, most viruses have also evolved means to inhibit the prolonged activation of the DDR during infection by inhibiting various proteins involved in the response [55].

Other viruses, or viral gene products have also been implicated in directly increasing NKG2DL expression, including: adenovirus serotype 5 (Ad5) E1A oncogene, which binds to a transcriptional co-adaptor protein p300, resulting in increased NKG2DL expression in mice and human tumour cells [44]; the HIV-1 viral gene product Vpr specifically increases expression ULBP1 and ULBP2 on CD4^+^ T cells by activating ATR [42]; influenza virus increases surface expression of ULBP1, 2 and 3 on infected dendritic cells [56]; and ectromelia virus (ECTV) induces MULT1 expression in murine embryonic fibroblasts [57].

Viruses modulate cell signalling pathways upon infection to enable productive replication and biosynthesis, while preventing the host cell from undergoing apoptosis. One important pathway which is commonly hijacked by many viruses is the PI3K-Akt-mammalian target of rapamycin (mTOR) pathway, whereby, activation promotes cell survival, pro-viral metabolism and biosynthesis [46,58]. Several viruses have been shown to activate PI3K, including adenovirus [48], HSV-1 [59], human cytomegalovirus (HCMV) [47] and murine cytomegalovirus (MCMV) [60]. MCMV-induced PI3K activation has been demonstrated to increase expression of RAE-1 ligands in mouse cells [60]. However, PI3K activation alone was not sufficient to induce expression without viral infection, suggesting other additional signals and virus-induced stresses were also required for NKG2DL expression [60].

## 4. Virus Immune Evasion

Much of our understanding of how cell biology and immunology works has been gained from studying how viruses exploit, inhibit, or subvert these mechanisms for their own gain. Investigations into the viral attempts to inhibit the NKG2D system to achieve immune evasion have revealed the importance of the NKG2D response and helped to uncover various mechanistic details. Comparing the evolution of viruses from a range of distinct viral families reveals that many have independently converged on interfering with the NKG2D immune response [61].

Some viruses result in persistent infections, such as those from the *Herpesviridae* family including HCMV and HSV. Herpesviruses are enveloped dsDNA viruses with large genomes. The HCMV genome contains over 150 open reading frames (ORFs), enabling the virus to encode a wide array of proteins, each with distinct and specific functions. This large genome size endows herpesviruses with the potential to modulate the infected host cell, enabling immune evasion and persistent infections via a host of different viral proteins. In light of this, herpesviruses represent a significant proportion of the examples mentioned below in their strategies to interfere with the NKG2D immune response by using a variety of viral gene products [62].

### 4.1. Viral Proteins Use a Range of Strategies to Downregulate NKG2DL Expression

Much of the early work investigating the effects of viral modulation of NKG2DLs was conducted using cytomegaloviruses, MCMV and HCMV. MCMV has been shown to downregulate surface expression of each of the murine NKG2DLs through a variety of means: (1) MCMV glycoprotein gp40, encoded by the gene *m152*, resulted in the specific downregulation of surface expression of RAE-1α, β, δ, ε and γ [63]; (2) a protein encoded by MCMV gene *m155* was found to target H60 for lysosomal degradation, thus reducing surface expression [64]; (3) an MCMV glycoprotein encoded by gene *m145* downregulates MULT1 [65]; (4) fcr-1 protein, encoded by the gene *m138,* rapidly decreased surface expression of both MULT1 and H60 [66].

Early studies investigating the immune evasion mechanisms of HCMV demonstrated that HCMV membrane glycoprotein UL16 is capable of binding to MICB, ULBP1 and ULBP2, and that exogenous addition of soluble UL16 was able to block the binding of these ligands to the NKG2D receptor on NK cells [13]. Further investigations demonstrated enhanced NK cell-mediated killing of fibroblasts infected with UL16-deleted HCMV compared to the wild type virus, and was attributed to the downregulation of surface MICB, ULBP1 and ULBP2 [67]. Two studies by Welte et al. and Dunn et al. later demonstrated the mechanism of HCMV UL16-mediated downregulation of MICB, ULBP1 and ULBP2. They showed that UL16 is retained in the ER and cis-Golgi apparatus membranes, whilst simultaneously sequestering the NKG2DLs intracellularly by binding via the UL16 ectodomain [68,69]. Wu et al. were able to demonstrate that the intracellular retention of MICB was due to a tyrosine-based motif in the cytoplasmic domain of UL16, and deletion of this motif restored MICB trafficking to the cell surface [70].

Beyond UL16, HCMV also has other mechanisms to downregulate NKG2DLs. HCMV protein UL142 blocks surface expression of full-length MICA protein, again by retaining it in the cis-Golgi apparatus [71]. Interestingly however, not all alleles of MICA were affected equally. The MICA*008 allele has a truncated transmembrane region and has no cytoplasmic tail, masking it from UL142 binding and enabling surface expression in HCMV-infected cells [25,72]. This suggests a possible evolutionary pressure for this mutant allele to be selected for human resistance to HCMV infection, as MICA*008 is the most prevalent allele in several populations, including North American Caucasians [24]. However, recently evidence has emerged to suggest HCMV has since co-evolved alongside the MICA*008 allele, as the US9 protein has been found to specifically target MICA*008 for proteasomal degradation [73].

After performing a genome-wide screen for other HCMV proteins that may interact with NKG2DLs, Fielding et al. further identified a number of proteins in the US12 family [74,75]. They revealed that US18 and US20 were capable of targeting MICA for lysosomal degradation, and functioned either independently or together in concert [74]. They also reported that US12, US13 and US20 were involved in downregulating MICB and ULBP2 [75].

Other herpesviruses, such as HSV-1 and varicella-zoster virus (VZV), also downregulate NKG2DLs, although precise mechanistic details remain uncertain. For example, VZV upregulates MICA, but downregulates ULBP2 and ULBP3 surface expression, whereas HSV-1 decreases total MICA, ULPB2 and ULBP3 protein, and downregulates surface ULBP1 surface expression only [76]. However, Schepis et al. reported a downregulation in surface expression of MICA and ULBP2 only, with total protein remaining unchanged, and attributed this to a late viral gene product retaining the proteins intracellularly [77]. The discrepancies observed here may be due to the different cell lines tested, as Campbell et al. noted similar decreases in ARPE-19 epithelial cells, human foreskin fibroblasts and 293T human embryonic kidney cells, whereas Schepis et al. used tumour cell lines including U373 astroglioma cell line and HeLa epithelial tumour cell line.

Human herpesvirus (HHV)-7 U21 gene product has been demonstrated to bind and direct ULBP1 for lysosomal degradation, and downregulate surface MICA and MICB expression via an unknown mechanism [78]. Similarly, an unknown early viral gene product of HHV-6 downregulates surface MICB, ULBP1 and ULBP3 by targeting them for degradation [79]. Meanwhile, Kaposi’s sarcoma-associated herpesvirus (KSHV) expresses the protein K5 which has E3 ubiquitin ligase activity, resulting in ubiquitinylation of MICA (and other immunoreceptors including MHC class I) and the redistribution of MICA away from the cell surface and into intracellular compartments [80].

Other viruses have also evolved proteins to downregulate NKG2DL expression. Hepatitis C virus (HCV) non-structural protease NS3/4A has been implicated in reduced MICA and MICB expression, although the mechanism remains unclear [81].

Adenovirus also uses proteins to enable immune evasion. Although the Ad5 early gene E1A alone is capable of inducing expression of NKG2DLs when expressed by human cancer cells [44], Ad5 also has evolved other mechanisms to negate this effect and avoid NK cell killing. The Ad5 E3/19K gene not only downregulates HLA-1 during infection, but has also been demonstrated to directly cause retention of MICA and MICB in the ER, thus countering the immunogenic effects of the E1A gene and enabling immune evasion [82].

Hepatitis B virus (HBV) uses viral proteins to downregulate NKG2DLs via yet another mechanism. The HBV protein HBx promotes increased expression of the transcription factors GATA-2 and GATA-3 and forms a trimeric protein complex with HBx and GATA-2/3. This protein complex then binds to the promoter region for MICA and MICB and represses transcription [83]. Additionally, the HBV protein HBc also binds to CpG islands in the MICA/B promoters to further repress MICA/B transcription [83].

While the HIV-1 Vpr protein increases ULBP1 and ULBP2 expression in infected CD4^+^ T cells via induction of DNA damage sensors [42], HIV-1 also expresses a protein called Nef which subsequently downregulates MICA, ULBP1 and especially ULBP2 [84]. The Nef protein has already been implicated in its role in directing various immune receptors away from the cell surface such as CD4, CD28 and MHC class I molecules [85], however the precise mechanism for how Nef downregulates surface NKG2DLs appears to differ compared to other surface receptors [84].

Vesicular stomatitis virus (VSV) has been shown to dramatically increase MICA mRNA expression upon infection of Jurkat T cells; however, surface MICA protein expression is decreased on infected Jurkat T cells and melanoma cell lines [86]. Investigation into the possible mechanism of this MICA protein downregulation revealed that it was not dependent on: the matrix (M) protein, which is responsible for VSV immune evasion of type I interferon (IFN); or VSV-mediated global inhibition of host translation; or intracellular retention of MICA, as seen in HCMV-infected cells [86]. Although the exact mechanism and protein responsible could not be elucidated, the authors concluded the downregulation likely occurs at an early post-transcriptional level [86].

The sheer number and variety of examples outlined above of different viral proteins capable of limiting NKG2DL expression highlights the importance of evading the NKG2D immune response for viruses. Herpesviruses, in particular, represent a large proportion of these examples, with multiple distinct viral proteins responsible for limiting NKG2DL protein stability or surface localisation. The diversity of examples suggests that intracellular retention or degradation of NKG2DLs is a highly effective immune evasion strategy.

### 4.2. Viral Strategies to Evade Pattern Recognition Receptors

Double-stranded RNA (dsRNA) or dsDNA viral genomes present in the cytoplasm are sensed by the pattern recognition receptors (PRRs) such as retinoic acid-inducible gene (RIG)-1, melanoma differentiation-associated protein (MDA)-5 and cyclic-GMP-AMP synthase (cGAS). These PRRs can trigger increases in NKG2DL expression upon infection with cytoplasmic DNA viruses, such as vaccinia virus (VV). To counter this, the VV gene *EL3* encodes a dsRNA binding protein, which shields dsRNA produced during infection from recognition by PRRs. Avoiding detection by PRRs is a surprisingly effective mechanism, as infection of fibroblasts with *EL3*-deleted VVs shows increased surface NKG2DL expression and sensitises them to NK cell lysis [87].

HIV-1 also has an indirect method of avoiding detection by the host cell and thus preventing the expression of NKG2DLs. Apolipoprotein B-editing complex 3G (APOBEC3G or A3G) is an antiviral factor which forms part of the innate immune defence system against viruses, by deaminating cytidine residues in viral genomes and causing viral hypermutation and inactivation. The activity of A3G induces the DDR in infected cells, thus triggering NKG2DL expression and sensitivity to NK cell lysis [88]. To counteract this however, the HIV-1 viral infectivity factor (Vif) targets A3G for proteasomal degradation and thus prevents DDR-induced NKG2DL expression [88].

### 4.3. Viruses Can Use miRNAs to Regulate NKG2DL Expression at a Post-Transcriptional Level

The 3′ UTRs of MICA and MICB mRNAs have conserved sites that are targeted by cellular miRNAs that destabilise the transcripts and trigger mRNA degradation. miRNA destabilisation is believed to be part of the normal regulation of MICA/B expression in healthy cells [37]. Interestingly, HCMV also exploits this system of regulation. HCMV miRNA-UL112 targets the same 3′ UTR of MICA and MICB mRNA transcripts as the cellular miRNAs, resulting in destabilisation and degradation of MICA and MICB mRNAs and preventing protein translation [37,89]. Other herpesviruses like KSHV and EBV also express miRNAs, miR-K12-7 and miR-BART2-5p respectively, that target the 3′ UTR of MICB transcripts similarly to HCMV [90]. These miRNAs share no sequence homology between HCMV, KSHV and EBV however, and bind to different sites within the 3′ UTR, suggesting a degree of convergent evolution between different herpesviruses to target MICB [90].

Self-defence using miRNAs targeting NKG2DLs is also observed in other families of virus. For example, human polyomaviruses (PyVs) JCV and BK strains both express an identical miRNA, miR-J1-3p, which reduces surface ULBP3 expression [91]. However, the authors reported that the ULBP3 mRNA transcripts were not degraded, suggesting an alternative post-transcriptional means of repression, such as translation inhibition.

HSV-1 has developed an alternative, indirect method of using miRNAs to downregulate NKG2DLs at a post-transcriptional level. HSV-1 miR-H8 does not bind to NKG2DL mRNA transcripts directly; instead, it targets mRNA transcripts for one of the key proteins involved in covalently attaching proteins to GPI anchors at the cell surface [92]. Therefore, by preventing translation of part of the GPI-anchoring machinery, it reduces surface expression of some of the GPI-anchored NKG2DLs such as ULBP2 and ULBP3 [92].

### 4.4. Virus-Mediated Shedding of NKG2DL

HCMV miRNAs have been further implicated in downregulation of surface NKG2DL, albeit via a different and indirect mechanism. HCMV miRNA-US25-2-3p binds to the 3′ UTR of mRNA transcripts encoding for tissue inhibitor of metalloproteinases-3 (TIMP-3) and leads to degradation of the mRNA. TIMP-3 is an inhibitor of metalloproteinases on the surface of cells such as a disintegrin and metalloprotease (ADAM) 17 and matrix metalloprotease (MMP) 14. The subsequent reduction in TIMP-3 protein results in enhanced activity of such metalloproteases, leading to an increased cleavage and shedding of MICA and MICB from the surface of HCMV infected cells [93].

Patients with chronic HIV-1 infections have been reported to have increased levels of soluble NKG2DLs in their sera, and HIV-1 infection of CD4^+^ T cells in vitro lead to increased MICA, MICB and ULBP2 surface expression but also increased protein shedding, [94]. Furthermore, the shedding of NKG2DLs in HIV-1 infected lymphocytes could be prevented with the addition of MMP inhibitors, implying possible a link between viral upregulation of cellular MMPs [94].

### 4.5. Virus-Mediated Immune Subversion

While many viruses have evolved mechanisms to downregulate the NKG2DLs on the infected host cells to achieve immune evasion, some viruses instead use other mechanisms to avoid destruction. For example, zoonotic orthopoxviruses (ZPXVs), such as cowpox and monkeypox have evolved an entirely alternative strategy to evade NKG2D mediated detection. These ZPXVs express a highly conserved orthopox MHC class I-like protein (OMCP), which is secreted from infected cells and antagonises NKG2D receptors themselves with high affinity [95]. Alternatively, HCV non-structural protein NS5A stimulates monocytes to shift their cytokine profile via binding to Toll-like receptor (TLR4), increasing IL-10 whilst decreasing IL-12 secretion levels. The increased IL-10 secretion leads to a concomitant increase in transforming growth factor (TGF) β, which downregulates NKG2D receptor expression on circulating NK cells [96].

Other cytokines produced in response to viral infection have also been identified. Muntasell et al. revealed that peripheral blood mononuclear cells (PBMCs) infected with HCMV in vitro caused a selective decrease in NKG2D receptor on NK cells [97]. However, this downregulation was found to be transient, with normal expression returning 7 days after infection. Interestingly, this effect could be abrogated by antagonising type I IFN, IL-12 or IFN-γ, indicating that these proinflammatory cytokines were responsible. The authors suggested that this cytokine-mediated downregulation of NKG2D receptor may be part of a physiological negative feedback system to limit NK cell responses against healthy cells expressing NKG2DLs during inflammatory responses [97].

As mentioned above, elevated levels of soluble NKG2DLs in patient sera have been observed in HIV^+^ patients, attributed to shedding of ligands from HIV-1 infected lymphocytes [94]. Furthermore, in vitro experiments using plasma from HIV-1-infected patients caused a significant downregulation of NKG2D receptor on NK cells and CD8^+^ T cells obtained from healthy donors [94]. Compared to patients receiving highly active antiretroviral therapy (HAART) with lower soluble NKG2DL levels, naïve HIV^+^ patients with elevated soluble NKG2DL levels displayed reduced NKG2D receptor expression on circulating NK cells and CD8^+^ T cells [94]. These findings suggest that soluble NKG2DLs not only decrease target ligands on the infected cell surface, but also affect the capacity for an NKG2D-mediated immune response on a systemic level by downregulating NKG2D receptor. Following these findings, it not unlikely that shedding of MICA and MICB due to HCMV miRNA-US25-2-3p mentioned above [93] may also cause a systemic decrease in NKG2D receptor levels on immune cells, although this has not yet been investigated. The mechanism of soluble NKG2DL-mediated downregulation of NKG2D receptor expression will be discussed later in Section 6.2.

### 4.6. Evolution of Viral Immune Evasion has Converged on Disrupting the NKG2D System

The NKG2D system appears to be a critical component in immune control of viral infection, given the examples of multiple viruses from entirely distinct families deploying various strategies to prevent NKG2D signalling (Table 1). While some viruses directly reduce the expression of NKG2DLs of the infected host cell, others achieve the same results via different means instead, by targeting the NKG2D receptor itself either directly or subverting the immune response indirectly. These examples of convergent evolution by multiple related and unrelated viruses demonstrate the importance of the NKG2D system in controlling viral infection.

## 5. NKG2DL Expression in Transformed Cells

Early observations that tumour cell lines were killed by NK cells and T cells lead to the discovery of the NKG2D receptor and its ligands [6]. Various studies revealed tumour cells induced to express RAE-1 and H60 proteins resulted in tumour rejection in mice in an NK cell-dependent manner [18,98,99,100]. The importance of NKG2D immune surveillance has been made clear in NKG2D-deficient mouse models, which spontaneously developed cancer much more than wild type mice [101].

### 5.1. Tumours Upregulate NKG2DLs via Chronic DDR

Increased NKG2DL expression has been reported in many human cancers including leukaemia [102], colorectal carcinoma [103], hepatocellular carcinoma [104], melanoma [105], pancreatic carcinoma [106], breast carcinoma [107] and glioma [108].

Active DNA damage responses have been identified in cells undergoing the early stages of tumourigenesis in precursor lesions, including phosphorylated histone H2AX, p53 and kinases ATM and Chk2 [109,110]. These activated proteins are part of the DNA damage checkpoint, which can trigger cell cycle arrest or apoptosis of these cells undergoing the first stages of transformation to prevent tumourigenesis. Gasser et al. provided a mechanistic link between this DNA damage response and immune surveillance of premalignant lesions by the upregulation of NKG2DLs. By using various genotoxic stressors on both mouse and human non-tumour cell lines to induce an ATM/ATR-mediated DNA damage checkpoint, they demonstrated increased NKG2DL expression. This upregulation could be abrogated by specific inhibition of this DNA damage checkpoint. Furthermore, they suggest that established tumour cells sustain their increased NKG2DL expression due to chronic activation of the DNA damage checkpoint [32].

A further link between DNA damage in cancer cells and NKG2DL expression was later made with the observation that doxycyclin-inducible wild type (but not a DNA-binding mutant variant) p53 mediated ULBP1 and ULBP2 surface upregulation via increased levels of mRNA transcription in p53-null human non-small cell lung cancer cells [35].

Detection of cytosolic dsDNA by PRRs is critical in triggering the innate immune response towards cytosolic DNA, either of viral origin, or because of DNA damage associated with cancer cells. Cytosolic dsDNA is detected and bound to by the enzyme cGAS, which synthesises the soluble secondary messenger cyclic-GMP-AMP (cGAMP). cGAMP molecules then potently activate stimulator of interferon genes protein (STING), leading to phosphorylation of IFN regulatory factor (IRF) 3. Activated pIRF3 then causes transcriptional upregulation of type I IFN genes and triggers innate immune responses [111]. A study using mouse lymphoma cell lines demonstrated that the DDR in cancer cells resulted in cytosolic dsDNA, which activated the cGAS-STING pathway, triggering an IRF3-dependent induction of RAE-1 ligands [112].

### 5.2. NKG2DLs Are Upregulated in Tumours Through Deregulation of the Cell Cycle and Activated Cell Signalling

Excessive and dysregulated cell cycle entry is a feature of cancer cells. E2F transcription factors play a crucial role in regulating the transition from G1 phase to S phase and entering the cell cycle. Studies using proliferating mouse cancer cells, as well as normal primary murine fibroblasts induced to proliferate, and other highly proliferative tissues demonstrated that the proliferative signals in these cells induced increased transcription of *Raet1e* [113]. This increased transcription was dependent on E2F transcription factors, which have binding sites in the *Raet1e* promoter [113].

The PI3K pathway is a key regulator of cell survival and proliferation, and is commonly activated in the early stages of tumourigenesis [114]. PI3K activation is involved in maintaining sustained expression of RAE-1 and MULT1 in transformed mouse cell lines [60]. However, the activation of the PI3K pathway alone was not sufficient to induce expression in cells, suggesting other factors also influenced expression [60]. In line with this evidence, c-Myc activity (downstream of PI3K pathway) is involved in NKG2DL expression during early stages of tumourigenesis in a spontaneously developing murine lymphoma model [115].

As mentioned earlier, UVB stress-induced EGFR signalling can cause MICA, MICB and ULBP2 upregulation in human epithelial cells [36]. Given the EGFR signalling pathway is commonly hyperactivated in human cancers, and the positive correlation between EGFR and NKG2DL expression in human carcinomas [36]. This may highlight another mechanism in which cancer cell phenotypes induce NKG2DL expression.

## 6. Cancer Strategies to Evade NKG2D

As observed with a variety of different viruses, there is also a significant selective pressure for immune evasion in to cells undergoing malignant transformation, and effective immune evasion is now recognised as one of the hallmarks of cancer [1].

Given this selective pressure applied to cancer cells, it is unsurprising that, similarly to several viral infections, many cancers also acquire mechanisms to promote immune escape by modulating the NKG2D response. For example, experimentally induced tumour cells were positive for RAE-1 protein expression in perforin-deficient mice, whereas expression was low or absent in WT mice [116], indicating a selection pressure exerted by the immune system for cancer cells to reduce or lose expression of NKG2DLs.

### 6.1. Loss of NKG2DLs May Be Involved in Metastatic Progression

Cancer stage progression has also been linked to NKG2DL expression in uveal melanoma patients. In a study by Vetter et al., half of the primary tumours sampled were positive for MICA/B, yet all metastatic lesions were absent for MICA/B, suggesting an involvement of the loss of MIC expression in tumour progression and metastasis [117].

### 6.2. Downregulation of NKG2Ds on Immune Cells by Cell-Bound and Soluble NKG2DLs

Groh and colleagues made an early observation in patients with a range of human epithelial tumours, including breast, lung, ovarian, colon and melanoma, that NKG2D receptor expression was significantly decreased on CD8^+^ tumour-infiltrating lymphocytes (TILs), NK cells and γδ T cells in patients with tumours positive for MIC [118]. By coculturing PBMCs with B cells transfected with MICA or MICB, they noted that surface NKG2D expression was significantly decreased on CD8^+^ T cells compared to PBMCs cocultured with un-transfected controls after 48 h. Additionally, they noted that total NKG2D receptor expression was decreased in these PBMCs, suggesting protein recycling from the cell surface and degradation. Subsequent treatments with bafilomycin A1 or chloroquine (inhibitors of lysosomal acidification and protein degradation) prevented the decrease in total protein. They concluded that surface NKG2D protein was recycled and degraded upon interaction with MICA or MICB positive cells, in a process of ligand-induced recycling in a manner similar to CD28 and TCR-CD3 complexes, following antigenic stimulation [119,120].

However, Groh and colleagues observed a similar decrease in surface and total NKG2D receptor expression in circulating PBMCs, not just those in the tumour, in patients with MIC-positive tumours. This observation ruled out the possibility that this downregulation was solely due to interactions with NKG2DL-expressing cancer cells. They subsequently discovered that the patients with MIC-positive tumours and low NKG2D receptor expression on TILs and PBMCs had soluble MIC (sMIC) A and sMICB in their blood sera, but patients with MIC-negative tumours did not. Treatment of CD8^+^ T cells with recombinant sMICA recreated the NKG2D receptor decrease [118]. This observation of epithelial tumours shedding NKG2DLs was one of the first immune evasion mechanisms of the NKG2D system to be discovered.

Shed NKG2DLs in the form of sMICA and sMICB have not only been observed in epithelial cancer patients, but also haematopoietic malignancies such as leukaemia [102]. Strikingly, in a comprehensive analysis of 205 leukaemia patients, all patient sera contained soluble NKG2DLs [121].

### 6.3. Mechanisms of NKG2DL Shedding by Tumour Cells

Investigations into the mechanisms of NKG2DL shedding have revealed this process is mediated by metalloproteases on the surface of the cancer cells, and that shedding can be inhibited with the addition of metalloprotease inhibitors [122]. Various metalloproteases have been implicated in NKG2DL shedding, particularly enzymes from the ADAM family, specifically ADAM10 and ADAM17 being responsible for MICA [123] and MICB cleavage [124]. Additional evidence suggests the thioreductase activity of ER protein 5 (ERp5) is also required for MICA shedding [125]. Furthermore, MICA and MICB shedding from a range of tumour cell lines was attributed to either or both ADAM10 and ADAM17, but the regulation of this process was different depending on the cancer cell lines tested [126]. Additionally, genotoxic stressors such as doxorubicin and melphalan have been demonstrated to upregulate ADAM10 activity on multiple myeloma cells, resulting in increased MIC shedding [127].

In contrast to the reported ADAM10 and ADAM17 activity for MICA/B shedding, MMP14 has been demonstrated to cause MICA shedding in prostate and breast cancer cell lines, independent of ADAM activity [128]. Additionally, studies on malignant glioma cells revealed that only ULBP2 was shed via ADAM10 and ADAM17 activity [129]. Similar observations have also been seen with glioma stem-like cells, with ADAM10- and ADAM17-dependent shedding of ULBP2 [130].

These discrepancies observed in the precise mechanisms and enzymes involved in NKG2DL shedding are likely to be due to the heterogeneities between the various cancer cells and cell lines used in the experimental models, as well as the allelic heterogeneity within the population. However, it does reveal a degree of complexity in the regulation of NKG2DL shedding.

Interestingly, the most common allele for MICA, MICA*008, which features a different transmembrane region and a truncated cytoplasmic tail compared to other alleles, is shed in a protease-independent manner. Instead of being shed by proteolytic cleavage at the cell surface, MICA*008 is released from cancer cells within the membranes of exosomes. These MICA*008-containing exosomes are still able to downregulate the NKG2D receptor on NK cells [131]. The selection of an allele for MICA in the population, which is resistant both to viral MICA-binding proteins as well as proteolytic cleavage by host cell metalloproteases upregulated in cancers, may highlight another example of human evolution to bypass cancer and virus-mediated immune evasion. However, cancer shedding of MICA*008 via exosome secretion appears to be one way in which cancers have adapted to circumvent this.

### 6.4. Soluble NKG2DLs Affect Other Immune Cell Types

While sMIC has been shown to decrease surface expression of NKG2D receptors on CD8^+^ T cells and NK cells, Xiao et al. demonstrated that mouse bone marrow cells differentiated into myeloid-derived suppressor cells (MSDCs) when treated with sMIC, while macrophages differentiated into ‘alternatively’ activated immunosuppressive macrophages [132]. This suggests tumour shedding of MIC not only directly enables immune evasion on a systemic level by global downregulation of NKG2D receptors on PBMCs and TILs, but also helps to establish an immunosuppressive tumour microenvironment through the repolarisation of myeloid cells.

Interestingly, Deng et al. provided evidence in contrast to the concept that soluble NKG2DLs exclusively suppress NK cell activity by downregulating NKG2D receptor [133]. These new findings led to the proposal that soluble MULT1 (sMULT1) actually enhanced NK cell-mediated tumour rejection. This was attributed to high affinity sMULT1 being able to bind to NKG2D receptors on NK cells, out-competing the binding of lower affinity NKG2DLs expressed by tumour-associated myeloid cells which would trigger NKG2D receptor downregulation, similarly to sNKG2DLs [133].

### 6.5. Tumours Use miRNAs to Downregulate NKG2DL Expression at the Post-Transcriptional Level

The use of miRNAs to regulate the stability of MICA/B mRNA transcripts has already been mentioned in the context of healthy and stressed cells, and that HCMV miRNA-UL112 exploits this system to enable immune evasion. This strategy has also been implicated in cancer immune evasion. Cellular miRNAs such as miR-20a, miR-93 and miR-106b which bind to the 3′ UTRs of MICA/B transcripts are upregulated in many human cancers, and are responsible for the downregulation in MICA and MICB protein [37]. Interestingly, some of these same cellular miRNAs had already been identified as cancer-associated miRNAs, with involvement in other pro-tumour processes in cancers, including breast cancer [134] and leukaemia [135]. Various other examples include: miR-10b, a metastasis-associated miRNA, which targets MICB transcripts in breast cancer cell lines [136]; miR-20a targets MICA and MICB transcripts, and also inhibits the MAPK/ERK pathway to downregulate ULBP2 in breast cancer [137]; miR-34a and miR-34c downregulate ULBP2 [38]; miR-93, miR-20a and miR-106b reduced expression of MICA, MICB, ULBP2 and ULBP3 in glioma cells [138]; and miR-889 which targets MICB transcripts in hepatocellular carcinoma cells [139]. Interestingly, both miR-20a [137] and miR-889 [139] expression decreased upon treatment with histone deacetylase (HDAC) inhibitors, resulting in an increase in NKG2DLs. These findings may provide some mechanistic insight as to why HDAC inhibitors increase cancer cell susceptibility to NK cell lysis [140].

### 6.6. Other Strategies Used by Tumours Subvert the NKG2D Immune Response

Other mechanisms to downregulate NKG2DL expression by cancer cells have also been observed. Isocitrate dehydrogenase (IDH) is mutated in many glioma patients and results in the production of the oncometabolite 2-hydroxyglutarate (2-HG). 2-HG inhibits histone demethylases and can cause epigenetic hypermethylation [141]. In primary human glioma stem-like cell lines, IDH mutated cells led to NKG2DLs becoming transcriptionally silenced as a result of 2-HG accumulation [142].

While some tumours intervene in NKG2DL expression before the protein translation stage, examples from melanoma cell lines and patient-derived metastases revealed a strategy more similar to that observed by viruses: intracellular retention of NKG2DL proteins [143]. These cells lacked surface MICA expression but still expressed intracellular protein, which was accumulated in the ER before being transported back to the cytoplasm for proteasomal degradation [143].

Cytokines in the tumour microenvironment can also influence immune evasion via the NKG2D system. TGF-β expressed by glioma cells enables immune evasion from the NKG2D system in several ways: (1) downregulation of NKG2DLs on the surface of glioma cells by inhibiting MICA, ULBP2 and ULBP4 transcription [144,145], (2) increased expression of inhibitory receptor CD94/NKG2A to prevent NK cell activation [144], and (3) downregulation of NKG2D receptors on CD8^+^ T cells and NK cells [146]. These findings suggest that TGF- β produced by glioma cells acts in both an autocrine and paracrine modality to mediate immune evasion from NK cells and CD8^+^ T cells. Additionally, Eisele et al. demonstrated by immunohistochemistry of gliomas that MICA and ULBP2 expression is inversely correlated with WHO grade of malignancy, although tumour expression was significantly higher than normal brain [145]. Furthermore, although TGF-β signalling increases metalloproteinase expression on glioma cells [147], the increased shedding of MICA and ULBP2 by glioma cells was not due to TGF-β-dependent metalloproteinases [145].

Further examples of cancer cells subverting the anti-cancer immune response by secretion of soluble factors have been observed in glioblastoma patients. Glioblastoma cells were found to express lactate dehydrogenase (LDH) isoform 5, which induced the expression of NKG2DLs on autologous tumour infiltrating myeloid cells and circulating monocytes in patients, as well as on monocytes isolated from healthy individuals [148]. These myeloid cells expressing NKG2DLs behaved similarly to soluble ligands upon interaction with NK cells and resulted in NKG2D receptor downregulation [148].

Surprisingly, pro-inflammatory IFNs appear to play a role in NKG2DL expression. Bui et al. reported that either IFN-γ or IFN-α reduced H60 expression on 3′methylchloanthrene sarcomas in mice [149]. Additionally, Zhang et al. reported IFN-α increased MICA expression, meanwhile IFN-γ decreased MICA expression in various human cancer cell lines, including cervical cancer and erythroleukemia, by promoting MMP-mediated shedding of MICA [150]. Furthermore, IFN-γ decreased transcription of MICA, ULBP1 and ULBP2 in patient-derived melanoma and glioma cell lines in a dose- and time-dependent fashion [151]. Investigations into the mechanism of this IFN-γ-mediated downregulation revealed that IFN-γ induced the expression of miR-520b, a miRNA that targets the 3′ UTR of MICA mRNA transcripts in a range of cancer cell lines including melanoma, HeLa, breast and colorectal [152]. It has been speculated that the downregulation of NKG2DLs in response to IFN-γ may be a regulatory mechanism that allows switching from innate immune surveillance via NKG2DLs to adaptive immune responses via MHC and T cells [149].

## 7. Viral and Cancer Immune Evasion Strategies Converge on the NKG2D Response

**Figure 2:**
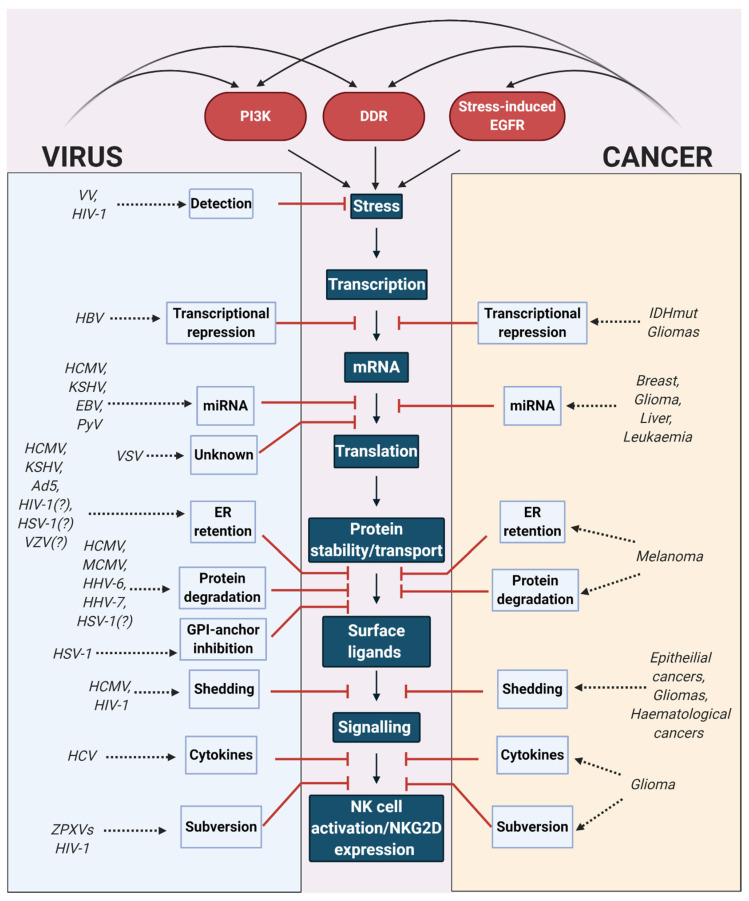
Converging evolution of viruses and cancers to interfere with the NKG2D immune response. Viruses and cancers induce various signalling pathways that induce natural killer group 2 member D ligand (NKG2DL) expression, including the phosphatidylinositol 3-kinase (PI3K) pathway, DNA damage response (DDR) and stress-induced epidermal growth factor receptor (EGFR) signalling. Various viruses and cancers interfere with the key stages in NKG2DL expression, including detection of stress, transcription, mRNA stability, translation, protein stability and transport, surface protein levels, NKG2D signalling and immune cell subversion. Vaccinia virus (VV), human immunodeficiency virus-1 (HIV-1), hepatitis B virus (HBV), human cytomegalovirus (HCMV), murine cytomegalovirus (MCMV), Kaposi’s sarcoma-associated herpesvirus (KSHV), Epstein-Barr virus (EBV), polyomavirus (PyV), vesicular stomatitis virus (VSV), adenovirus serotype 5 (Ad5), herpes simplex virus-1 (HSV-1), varicella-zoster virus (VZV), human herpesvirus (HHV), hepatitis C virus (HCV), zoonotic orthopoxvirus (ZPXV). Diagram created with BioRender.com.

The focus of this review is to bring attention to the similarities between virus and cancer immune evasion via the NKG2D system. Several viruses from a wide range of viral families use strategies to interfere with the key stages in the pathway, ranging from avoiding initial detection by PRRs, to transcriptional repression, mRNA stabilisation, NKG2DL protein stability and cellular localisation, shedding from the protein surface and subversion of the subsequent immune response. Several different cancers also interfere with many of these critical stages, sharing a striking similarity with viral immune evasion (Figure 2).

Viruses can use an expansive toolkit to achieve immune evasion, using wholly new proteins or miRNAs acquired through countless generations of evolution that are well-suited to carry out their specific task. However, cancers must make use of a relatively limited toolkit in comparison to achieve the same goal, as they are constrained by a shorter evolutionary timeframe, and must rely on proteins or miRNAs already encoded in their genomes, or slight variations thereof. Therefore, cancers must find ways to manipulate their toolkit, turning tools which normally serve the cell, tissue, and organism, into tools which enable cancer survival. This observation is highlighted by the abundance and variety of viral proteins that are unique to those viruses, which directly bind to, interfere with, or degrade NKG2DLs. Meanwhile, cancers appear to have a reliance on the tools already available to them, albeit by dysregulating them, such as upregulating cellular miRNAs to destabilise mRNA transcripts, or by activating surface proteases to increase NKG2DL shedding. Given the differences in the tools available at their disposal, is perhaps surprising to see such a high degree of functional convergence between the immune evasion strategies of both viruses and cancers.

## 8. Strategies Employed by the Immune System to Counter Viral and Tumour Immune Evasion

In parallel to the selection pressures exerted on viral and tumour evolution, they impart their own selection pressure on the immune system, driven by the necessity to constantly adapt and counter immune evasion tactics employed by pathogens and cancers. While viruses and cancer cells develop ways to downregulate or subvert the NKG2D response, the immune system has developed various means to counteract.

For example, using a family of multiple different ligands that are all capable of binding to the NKG2D receptor endows certain advantages to the organism. It enables a degree of functional redundancy, in which various signals and pathways induce the expression of different ligands, all of which converge on binding to NKG2D and bringing about a similar immune response [153]. However, evidence is emerging to suggest that NKG2DLs are non-redundant, and that induced expression of certain ligands reflects a specific danger signal and threat [154]. For example, ULBP1 expression positively correlates with leukaemia sensitivity to NKG2D-mediated lysis by γδ T cells, and loss of ULBP1 conferred resistance to lysis, whereas MICA downregulation did not [155]. Although specific pathways may induce the expression of certain ligands, the pathways themselves tend to be shared by a general cellular phenotype that reflects danger: either stress, infection, or neoplastic transformation. Furthermore, ligand diversity makes total immune evasion as a result of viral gene products or a mutation during malignant transformation far more unlikely [153,154].

In addition to the range of different NKG2DLs, they are also some of the most polymorphic genes in humans. Different ligands, and even different alleles can change the extent of NKG2D receptor signalling. For example, the MICA-129Met variant has a far greater binding affinity to NKG2D compared to the MICA-129Val isoform, and results in stronger signalling and faster activation kinetics [22]. Conversely however, the ULBP0602 isoform has a greater binding affinity compared to other isoforms, but resulted in reduced NKG2D-mediated activation [23]. These findings suggest different polymorphisms of NKG2DLs in the population that differ in binding affinities may influence the intensity of the immune response towards the target cell.

Further evidence for the evolution of the NKG2D system being driven by viruses is the MICA*008 allele, which lacks the cytoplasmic tail and thus cannot be bound and downregulated by HCMV UL142 protein [25,72]. The prevalence of this allele in the human population may highlight the selective advantage of this allele in conferring resistance to HCMV infection.

## 9. Conclusions and Outlook

Cancer immunotherapy is a rapidly advancing field, with vast potential for using the patient’s own immune system, or genetically engineered immune cells to eradicate the tumours at multiple sites throughout the body. Given NKG2DLs are expressed by many different cancer cells, they appear as an attractive ‘pan-cancer’ antigen to target using immunotherapies, and could circumvent issues with other immunotherapies associated with tumour heterogeneity and loss of MHC class I expression in tumours. This strategy has already been pursued, such as by the use of NKG2D chimeric antigen receptor (CAR) T cells, with evidence of efficacy even in heterogeneous tumours in vivo [156]. Furthermore, NKG2D-targeted immunotherapies may combine advantageously with conventional therapies which further induce NKG2DL expression. For example, Weiss and colleagues demonstrated synergy between radiotherapy and NKG2D CAR T cells in murine glioblastoma models [157]. However, more understanding of NKG2DL expression in non-tumour cells is needed, given some on-target, off-tumour toxicities have been observed using NKG2D-CAR T cells [158].

Asides from using engineered immune cells, other strategies have been investigated to enhance natural innate immune responses towards tumour cells expressing NKG2DLs. Techniques to inhibit proteolytic shedding of NKG2DLs, using chemical inhibitors [159,160] or antibodies which block the proteolytic cleavage site [161], have been explored. Additionally, antibodies to neutralise sMIC, preventing NKG2D receptor downregulation have also been investigated [162]. These are particularly attractive strategies and may work together in concert with NKG2D CAR T cells or other NKG2D-targeting cell therapies.

Oncolytic virotherapy (OVT) is a rapidly developing area for cancer immunotherapy. OVT uses modified viruses that selectively infect and replicate within cancer cells, causing lytic cancer cell death [163]. Many different viruses are being investigated for use as OVT, including adenovirus [164], HSV-1 [165] and VSV [86]. A notable example being an oncolytic HSV-1, Talimogene laherparevec (T-VEC), which gained FDA approval in 2015 [166]. As well as their ability for direct tumour lysis [167], oncolytic viruses can also be used as gene therapy vectors, delivering therapeutic agents in situ in the tumour. OVT for the treatment of cancer represents a cross-over between viruses and cancers. Targeting shared strategies of viral and cancer immune evasion of the NKG2D immune response could therefore provide significant, or even synergistic efficacy. One specific example could be to use the oncolytic virus as a gene therapy vector to deliver small interfering RNA (siRNA) within the cancer cell, knocking down ADAM10 and ADAM17 expression, and thus prevent NKG2DL shedding. The cumulative effects of heightened NKG2DL expression normally associated with cancer cells, alongside virus-induced NKG2DL expression, with further siRNA-mediated inhibition of NKG2DL shedding could drastically increase the likelihood of immune detection and cancer cell lysis.

As the field of immunology ever advances and we understand more about the similarities between viral and cancer immune evasion, we may gain further insight into new potential strategies to exploit the NKG2D system for anti-cancer therapy. Furthermore, NKG2DL-targeting therapies used for cancer treatment may even have a possible benefit for antiviral therapy.

## Figures and Tables

**Table 1:** Summary of viral mechanisms of NKG2D immune evasion.

Virus	Immunoevasin	Mechanism	Reference
MCMV	gp40	Downregulation of surface RAE-1α-γ expression	[63]
	m155	Targets H60 for degradation	[64]
	m145	Downregulation of MULT1 surface expression	[65]
	Fcr-1	Downregulation of MULT1 and H60 surface expression	[66]
HCMV	UL16	Retention of MICB, ULBP2 and ULBP3 in ER and cis-Golgi apparatus	[13,67,68,69,70]
	UL142	Retention of MICA in cis-Golgi apparatus	[25,71,72]
	US9	Targets MICA *008 for degradation	[73]
	US12, US13, US18, US20	Target MICA, MICB and ULBP2 for degradation	[74,75]
	miR-UL112	Targets 3′ UTR of MICA and MICB transcripts, causing mRNA destabilisation	[37,89]
	miR-US25-2-3p	Downregulation of TIMP3, causing increasing shedding by ADAM17 and MMP14 Shedding	[93]
VZV	Unknown	Downregulation of ULBP2 and ULBP3 by unknown mechanism	[76]
HSV-1	Late viral gene product(s)	Downregulation of MICA, ULBP1, ULBP2 and ULBP3 (cell-dependent) at a post-translational stage	[76,77]
	miR-H8	Disrupts expression of GPI-anchoring machinery, reducing ULBP2 and ULBP3 surface expression	[92]
HHV-7	U21	Targets ULBP1 for degradation and downregulates MICA and MICB by unknown mechanism	[78]
HHV-6	Early viral gene product(s)	Targets MICB, ULBP1 and ULBP3 for degradation	[79]
KSHV	K5	Ubiquitinylates MICA, causing redistribution from surface	[80]
	miR-K12-7	Targets 3′ UTR of MICB transcripts, causing mRNA destabilisation	[90]
EBV	miR-BART2-5p	Targets 3′ UTR of MICB transcripts, causing mRNA destabilisation	[90]
HBV	HBx	Forms complex with GATA-2/3 to repress MICA and MICB transcription	[83]
	HBc	Binds to CpG islands in MICA/B promoters to repress transcription	[83]
HCV	NS3/4a	Decreases MICA and MICB by unknown mechanism	[81]
	NS5a	Stimulates immunosuppressive cytokine production and NKG2D receptor downregulation	[96]
Ad5	E3/19K	Retention of MICA and MICB in ER	[82]
HIV-1	Nef	Downregulation of MICA, ULBP1 and ULBP2	[84]
	Vif	Degradation of A3G, preventing DDR-mediated NKG2DL expression	[88]
	Unknown	Shedding of MICA, MICB and ULBP2 by host MMP shedding	[94]
VSV	Unknown	Unknown (post-transcriptional)	[86]
VV	EL3	Prevents detection of dsRNA viral genome from PRRs	[87]
PyVs (JCV and BK)	miR-J1-3p	Targets 3′ UTR of ULBP3 mRNA and prevents translation	[91]
ZPXV	OMCP	Antagonism of NKG2D receptor	[95]

Murine cytomegalovirus (MCMV), human cytomegalovirus (HCMV), varicella-zoster virus (VZV), herpes simplex virus-1 (HSV-1), human herpesvirus (HHV), Kaposi’s sarcoma-associated herpesvirus (KSHV), Epstein-Barr virus (EBV), hepatitis B virus (HBV), hepatitis C virus (HCV), adenovirus serotype 5 (Ad5), human immunodeficiency virus-1 (HIV-1), vesicular stomatitis virus (VSV), vaccinia virus (VV), polyomavirus (PyV), zoonotic orthopoxvirus (ZPXV).
